# Lymph node recurrence and re-excision after primary tumor resection of a histiocytic sarcoma of duodenal origin: a case report

**DOI:** 10.1186/s40792-022-01545-z

**Published:** 2022-10-01

**Authors:** Kohei Segami, Shinjiro Kobayashi, Masaki Hiwatari, Yuta Ogura, Masafumi Katayama, Satoshi Koizumi, Motohiro Chosokabe, Junki Koike, Takehito Otsubo

**Affiliations:** 1 Division of Gastroenterological and General Surgery, St. Marianna University School of Medicine, 2-16-1 Sugao, Miyamae-ku, Kawasaki, Kanagawa 216-8511 Japan; 2 Department of Pathology, St. Marianna University School of Medicine, 2-16-1 Sugao, Miyamae-ku, Kawasaki, Kanagawa 216-8511 Japan

**Keywords:** Histiocytic sarcoma, Pancreaticoduodenectomy, Duodenal tumor

## Abstract

**Background:**

Histiocytic sarcoma is a rare malignant tumor that is similar in characteristics to a mature histiocyte/macrophage and is a relatively new disease entity. In approximately one-third of cases, the site of origin is a lymph node; development from the gastrointestinal tract, spleen, soft tissue, and skin has further been reported. The tumor characteristics are not well-understood as reports on its clinical presentation and treatment are limited. We report a case of duodenal primary histiocytic sarcoma.

**Case presentation:**

An elevated lesion in the second part of the duodenum was detected in a 70-year-old woman during routine examination using upper gastrointestinal tract endoscopy. Blood biochemistry findings were normal for tumor markers. No abnormal findings were observed in the blood count and biochemical examination. Upper gastrointestinal endoscopy revealed a 20-mm elevated lesion with a slight depression in the center, opposite to the papilla of the descending duodenum. The biopsy showed erosions of the mucosal epithelium and inflammatory cell infiltration, but no evidence of malignancy. Ultrasound-guided endoscopy revealed an ischemic tumor of submucosal origin, and bowel biopsy suggested a histiocytic sarcoma. Distant metastasis and lymph node enlargement were absent on abdominal sonography, computed tomography, and magnetic resonance imaging. Duodenal segmental resection was performed. Immunostaining of the excised lesion was positive for CD68, CD163, CD4, CD5, CD15, and CD45 and negative for CD1a, CD21, CD34, MPO, and S-100 protein. Ki-67 positivity was approximately 20%. Based on these findings, the diagnosis of histiocytic sarcoma was confirmed. Ten months after the surgery, a lymph node recurrence in the dorsum of the pancreatic uncus was observed. No evidence of recurrence was found in any other part; hence, we performed pancreaticoduodenectomy. Pathological findings of the excised lymph node confirmed the recurrence of histiocytic sarcoma in the lymph node.

**Conclusions:**

This is the first reported case of a duodenal primary histiocytic sarcoma with recurrence in the lymph node after the primary resection. The patient was treated for recurrence by lymph node excision and pancreaticoduodenectomy.

## Background

Tumors arising from histiocytes have been defined using different terms in the past, such as histiocytic lymphoma, histiocytic medullary reticulosis, or monocytic sarcoma; however, the nomenclature remains evolving [1]. According to the World Health Organization classification of 2001, a histiocytic sarcoma (HS) is a malignant proliferative disease that includes one or more kinds of histiocytic markers, except those of dendritic cells; immunologically, their nature resembles a mature histiocyte [2]. The collective term “histiocytic sarcoma” for such tumors (except acute monocytic leukemia) is a relatively new disease entity [3]. Definitions and classifications have not been unified, and there are only a few reported cases of HS. Herein, we report a case of HS that originated in the duodenum; the patient developed a recurrence, which was resected.

## Case presentation

The patient was a 70-year-old woman with an insignificant medical and family history. She visited a primary care family doctor for a routine clinical examination and underwent an upper gastrointestinal tract endoscopy. An elevated lesion was detected in the second part of the duodenum, and the patient was referred to our hospital for further examination.

### Diagnostic findings

On physical examination, no significant abnormalities were observed. Moreover, blood test findings were normal for tumor markers: the cancer antigen 19-9 and the carcinoembryonic antigen levels were 17.8 U/mL (normal range, 0–37.0 U/mL) and 2.1 ng/mL (normal range, 0–5.0 ng/mL), respectively. No abnormal findings were observed in the blood count and biochemical examination.

**Fig. 1:**
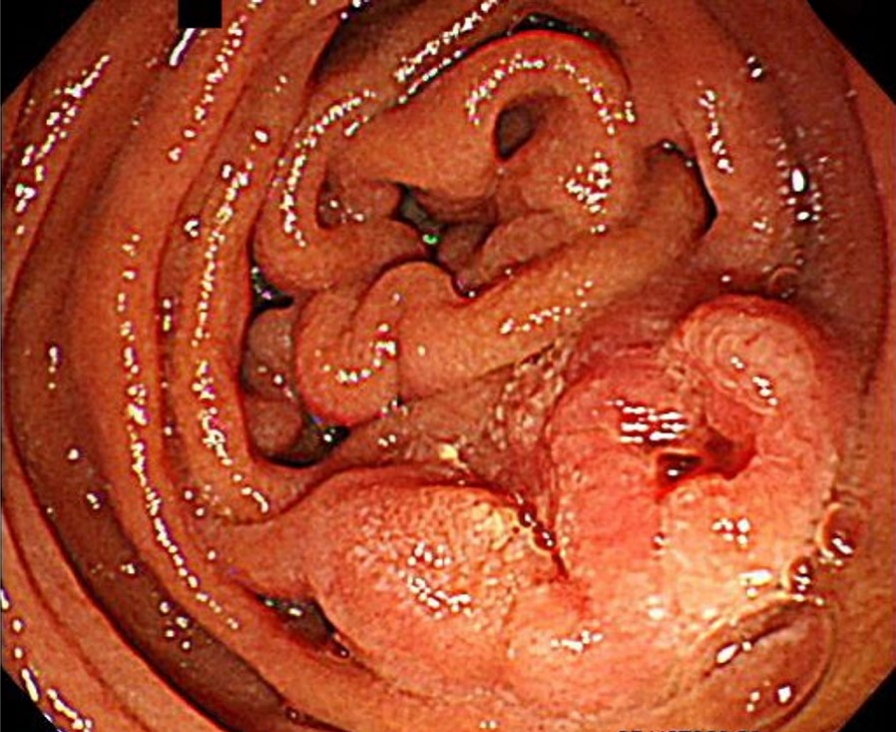
Endoscopy of the upper gastrointestinal tract. A 20-mm raised lesion with a slight depression in the center of the opposite site of the papilla of the second portion of the duodenum

Endoscopy of the upper gastrointestinal tract (Fig. 1) revealed a 20-mm raised lesion with a slight central depression in the duodenum, opposite the duodenum papilla of the second portion of the duodenum. We performed a biopsy, and no definite evidence of malignancy was identified. However, mucosal epithelial erosions and inflammatory cell infiltration were observed.

**Fig. 2:**
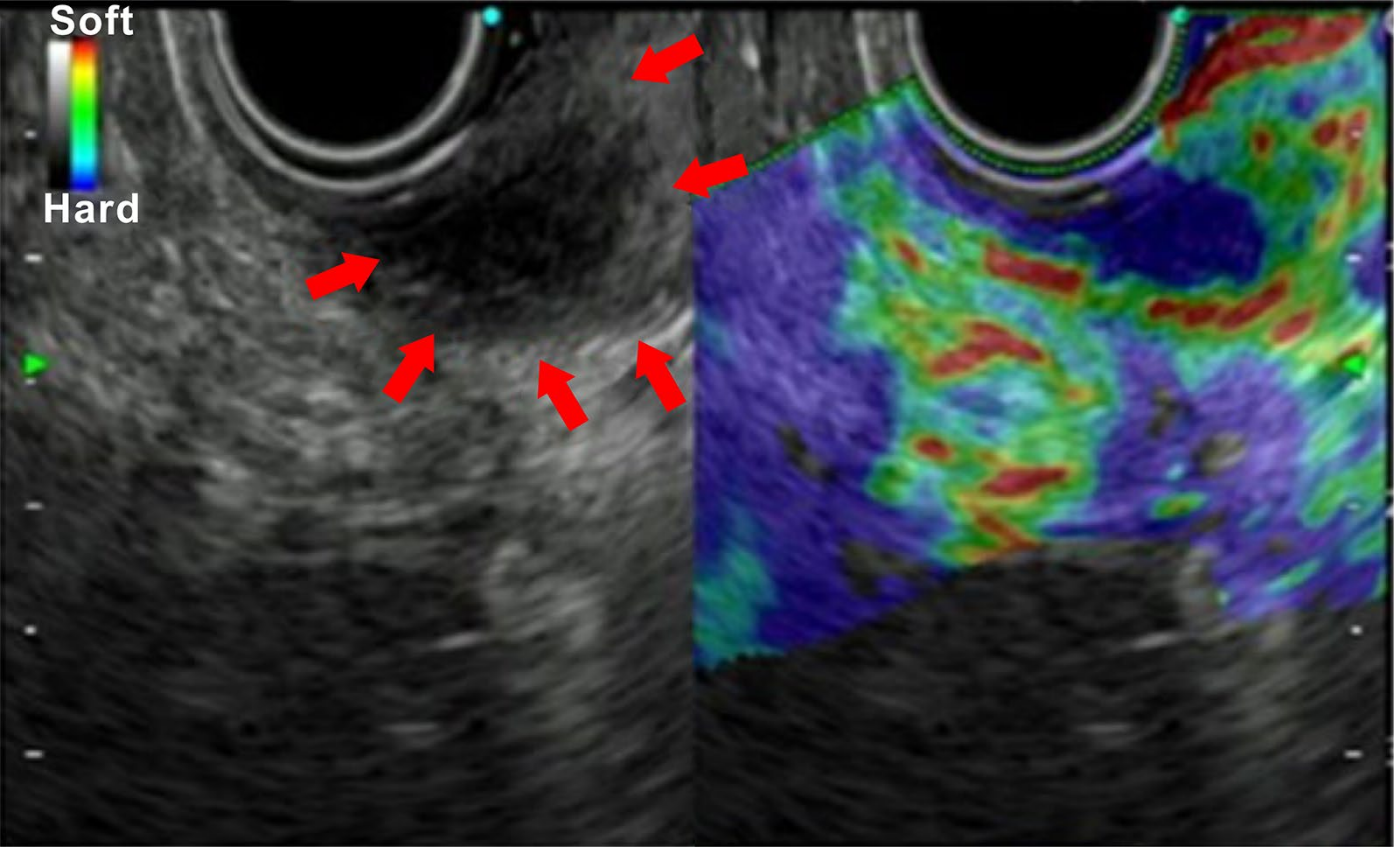
Ultrasound-guided endoscopic examination. An ischemic tumor of submucosal origin was suspected

Based on the ultrasound-guided endoscopic examination (Fig. 2), we concluded that the lesion was an ischemic tumor of submucosal origin, and that the depth of invasion was until the muscularis mucosa. Malignant findings were not identified in the previous biopsy; thus, we performed a bowel biopsy.

The bowel biopsy revealed tumor cells composed of proliferating atypical cells with a prominent nucleolus in a large nucleus and an acidophilic cytoplasm. Immunostaining was positive for CD68 and CD163 and negative for CD1a, CD21, HMB45, and S-100 protein. Abdominal sonography, computed tomography (CT), and magnetic resonance imaging (MRI) revealed no enlargement and distant metastasis to the lymph nodes. Positron emission tomography (PET) showed a mildly high maximum standardized uptake value (SUV-MAX) of 4.2 for the tumor site. Accumulation was absent in other sites apart from the tumor.

A diagnosis of primary HS of duodenal origin was made based on these findings, and surgery was chosen as the primary treatment. The tumor diameter was relatively small, and it was predicted that the tumor had invaded the muscularis. Moreover, no malignancy was found during imaging; hence, lymph node dissection was considered unnecessary. The duodenal segmental resection was limited to the duodenal papilla, and the tumor was enucleated with a secure margin.

### Postoperative pathological findings

**Fig. 3:**
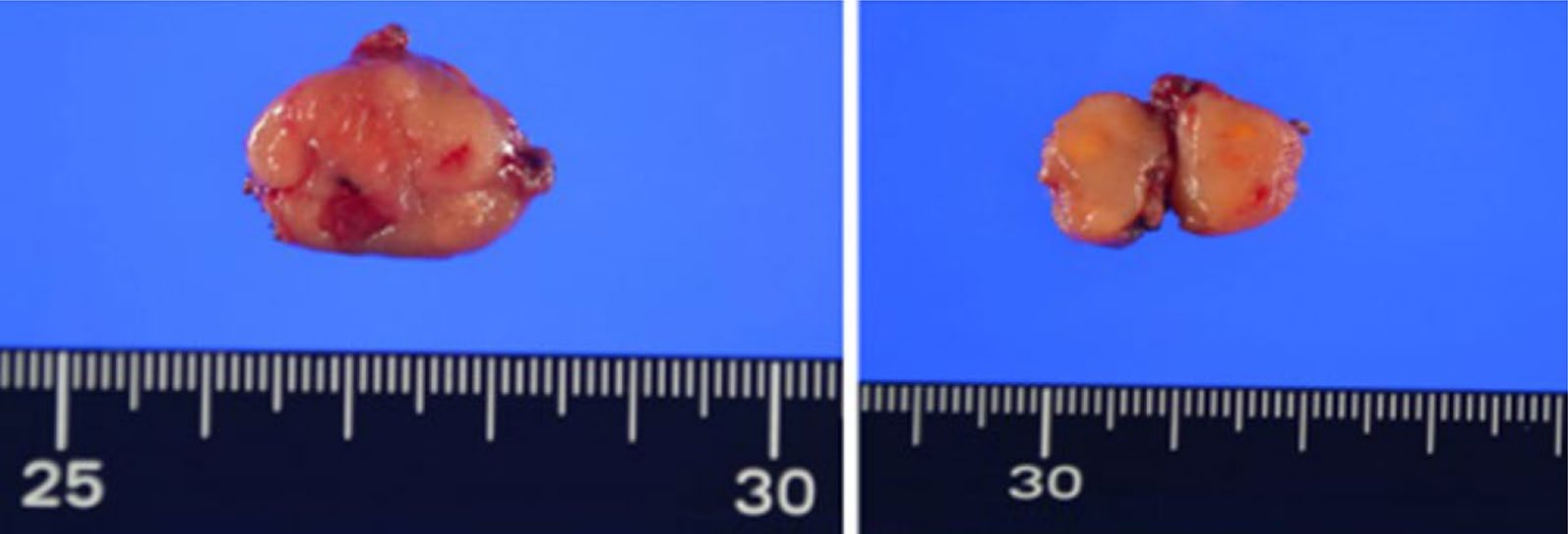
Postoperative pathological findings. The gross findings indicated a yellowish white mass lesion, 16 × 8 mm in size, histologically extending from the lamina propria to the muscularis mucosa

**Fig. 4:**
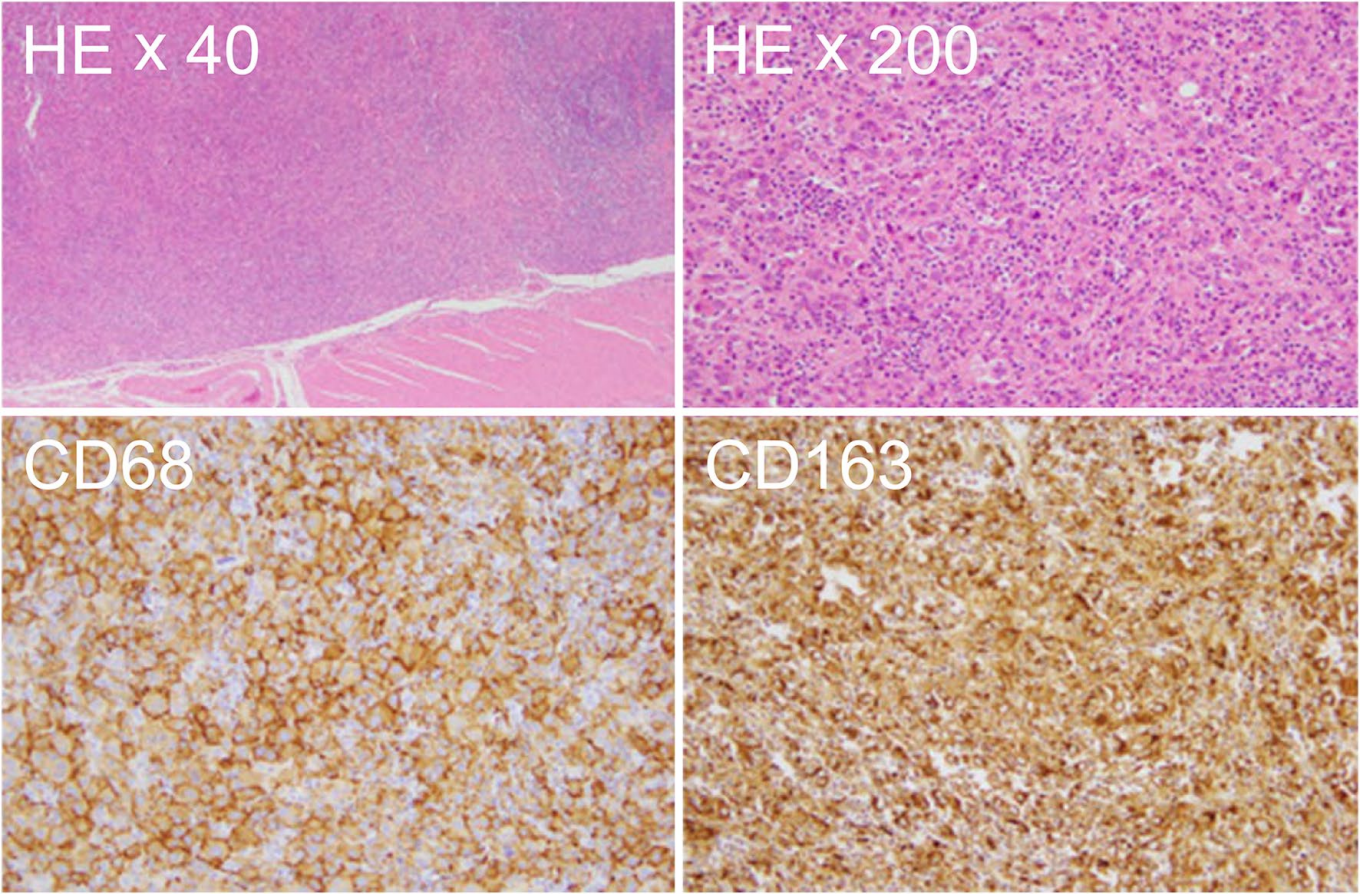
Microscopic findings. Significant hyperplasia of atypical tumor cells with acidophilic cytoplasm was seen. Immunostaining was positive for CD68 and CD163

The gross examination showed a yellowish white mass lesion, 16 × 8 mm in size, histologically extending from the lamina propria to the muscularis mucosa (Fig. 3). Additionally, significant hyperplasia of atypical tumor cells with acidophilic cytoplasms was detected. The stump of the resected specimen was tumor negative. Immunostaining was positive for CD68, CD163, CD4, CD5, CD15, and CD45 and negative for CD1a, CD21, CD34, MPO, and S-100 protein (Fig. 4). The Ki-67 positivity rate was approximately 20%. There was no evidence of lymphatic or venous invasion. Thus, the patient was diagnosed with HS.

### Postoperative course

The patient’s postoperative course was insignificant, and she was discharged on day 15 after surgery.

**Fig. 5:**
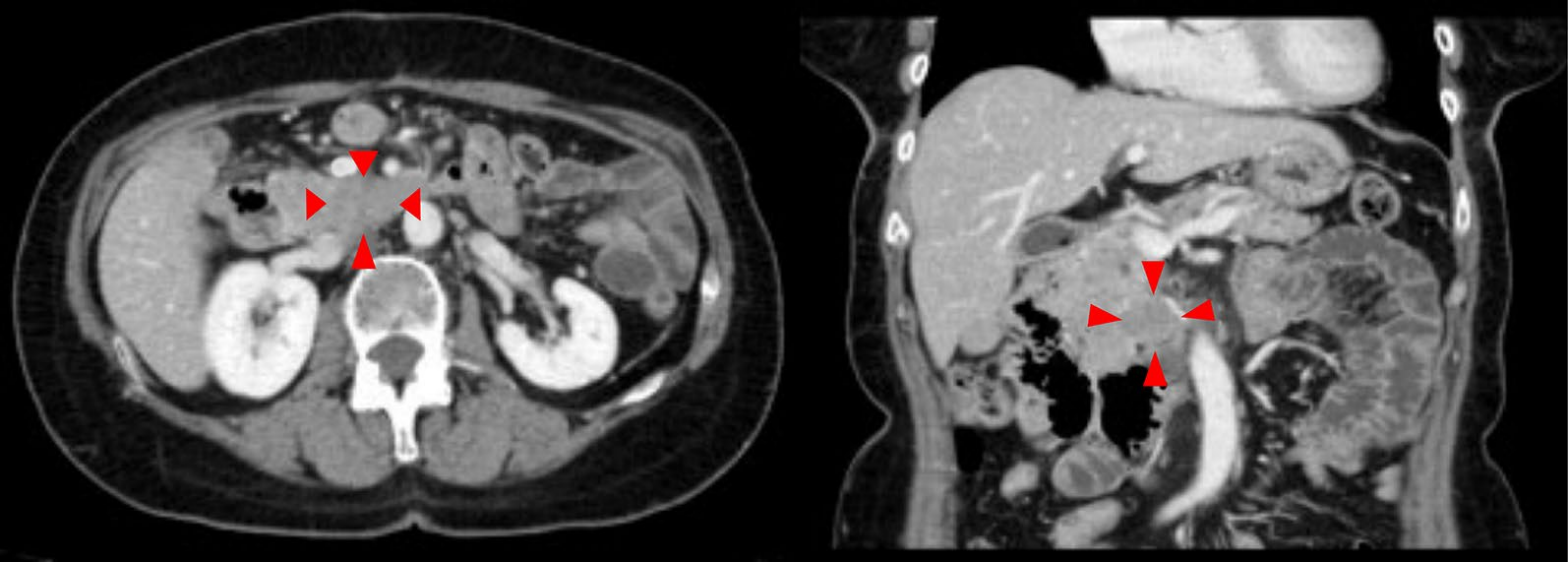
CT at the time of recurrence. Lymph node enlargement was detected in the dorsum of the pancreatic uncus 10 months after the surgery. CT, computed tomography

Ten months after the surgery, enlarged lymph nodes were detected in the dorsum of the pancreatic uncus on CT (Fig. 5). The lymph nodes showed hypointensity on diffusion-weighted MRI, and a recurrence of HS was suspected. The SUV-MAX on PET was 10.4. Since no other recurrent lesions (except these enlarged lymph nodes) were identified by various examinations, re-excision was chosen as the principal treatment, and a pancreaticoduodenectomy with lymph node dissection was performed. The lymph nodes around the superior mesenteric artery (SMA) and the hepatic artery were dissected.

### Histological examination of the re-excised tumor

Significant hyperplasia of blasts with an acidophilic cytoplasm, similar to that in the primary tumor, was noted during the pathological examination of the enlarged dorsal caput pancreatic lymph nodes; immunostaining was positive for CD68, CD163, CD4, CD5, CD15, and CD45 and negative for CD1a, CD21, CD34, MPO, and S-100 protein. Thus, we confirmed lymph node recurrence of the primary HS. Additionally, enlargement of the lymph nodes near the circumference of the SMA and the dorsal caput pancreatic lymph nodes were noted macroscopically; however, pathological examination revealed neoplastic tissue only in the dorsal caput pancreatic lymph nodes. There was no evidence of lymphatic or venous invasion. At the time of writing this case report, 11 months have passed since the recurrence; the patient is alive and has not experienced another recurrence.

## Discussion

Histopathologically, HS develops from a diffuse proliferation of cells with an acidophilic cytoplasm or cells with abundant foamy cytoplasm; dyskaryosis is strong, and imaging studies often reveal apoptotic cells and nuclear fission. Moreover, HS is often associated with reactive inflammatory cell infiltration. Because the cells in an HS can have an irregular, round, and spindle-shaped morphology, it may be difficult to distinguish an HS from a diffuse large B-cell lymphoma [4]. However, immunohistochemically, HS cells are positive for histiocytic markers such as CD68, CD163, CD204, and lysozyme; they express one or more histiocytoma markers, namely, Langerhans cell markers (CD1a and langerin) and follicular dendritic cell markers, but they do not express morphological markers (e.g., CD21 and CD35) and myelocytic markers (e.g., CD34 and myeloperoxidase) [2]. In the present case, significant hyperplasia of atypical tumor cells with a histologically acidophilic cytoplasm was found, and the patient was diagnosed with HS because the immunostaining was positive for CD68, CD163, CD4, CD5, CD15, and CD45 and negative for CD1a, CD21, CD34, MPO, and S-100 protein.

The epidemiological characteristics of HS are unclear as there are only a few reported cases. The age of onset varies widely, ranging from 20 to 89 years. [2] Approximately one-third of the cases have lymph nodes as the site of origin, followed by the gastrointestinal tract, spleen, soft tissue, and skin [5]. There are rare reports of HS occurring in the mediastinum and the central nervous system [3, 6]. The symptoms are non-specific, such as fever, malaise, weight loss, eruption, lymphadenopathy (neck and supraclavicular), hepatosplenomegaly, diarrhea, and bowel obstruction [7]; however, in the present case, the patient was asymptomatic.

In a report on five cases of splenic primary HS, thrombocytopenia was observed in all cases [8]. However, thrombocytopenia was observed neither in our case nor in cases where the tumor originated from the lymph nodes and other organs. Characteristic imaging findings have not yet been established. PET–CT can identify the lesion more precisely than sonography and CT, and it has been described that PET–CT is useful in estimating the efficacy of chemotherapy and discovering recurrence [9]. No standard guidelines have been established for therapy. Reports exist on the use of CHOP-based treatment (based on malignant lymphomas); however, it generally offers a poor prognosis [5]. Oka et al. have compared seven cases and reported a fatality with systemic metastases at diagnosis. However, the survival time was reportedly long in cases with HS either localized to the skin or other regions [10]. In addition, some reports have indicated that the tumor size has a prognostic value [11, 12]. However, these reports have not considered most cases in which death occurred, because the prognosis of patients with HS is poor, usually within 2 years from the initial diagnosis [13]. In particular, the prognosis is poorer for tumors originating in the central nervous system, with a median survival time of 4.5 months. [6]

Regarding gastrointestinal primary HS, we retrieved 15 cases reported from 2001 through 2021 using the keyword “histiocytic sarcoma” in PubMed and searching for “gastrointestinal tract”; thus, a total of 16 cases, including ours, were reviewed. To the best of our knowledge, this is the first reported case of an HS in the duodenum (Table 1). Among cases with a gastrointestinal primary HS, the median age at onset was 53.5 years (range, 20–89 years) and the sex ratio (male/female) was 3:5. The tumor size varied from 2–20 cm (mean, 7.1 cm; median, 6.0 cm). Stomachache was a common symptom, probably due to the relatively large tumor size at the time of initial diagnosis. The survival period, except in a case wherein the clinical course was unclear, was 2–120 months (median, 14 months). In a previous study, a patient with a small intestinal primary HS lesion had mesenteric lymphadenopathy around the lesion, which was resected along with the primary tumor. However, 18 months later, the patient experienced an intra-abdominal lymph node recurrence.^14^ In the present case, because the tumor was a small lesion limited to the duodenal descending limb during the time of the initial surgery, we chose duodenal segmental resection. However, the lesion recurred in the lymph nodes around the pancreatic uncus, 10 months after the surgery for the primary lesion. In this scenario, we could have initially chosen to perform a pancreatoduodenectomy, including a D2 dissection. However, because the prognosis of HS is poor, the prognosis was expected to remain the same even with a regional lymph node resection. Thus, the accumulation of evidence from future cases is crucial to establish the disease course and suitable therapy.

**Table 1:** Histiocytic sarcoma cases of the gastrointestinal tract reported since 2001

Case	Age (years)	Site	Initial symptoms	Tumor size (cm)	Treatment	Outcome	Follow-up	Recurrence
1	28	Stomach/jejunum	Abdominal pain	5.7	Surgery/chemotherapy	AW	36	−
2	58	Terminal ileum	Abdominal pain	8	Surgery	AW	12	−
3	89	Colon/stomach	Abdominal pain	12	Surgery	DOD	5	Sternum
4	40	Rectum	Hematochezia, abdominal pain, weight loss	7	Surgery	AW	21	−
5	27	Rectum	Hematochezia, abdominal pain, weight loss	9	Chemotherapy	AW	120	−
6	40	Anus	NA	2	Surgery	NA	NA	−
7	56	Small intestine	NA	NA	Surgery/chemotherapy	DOD	60	NA
8	46	Small intestine	NA	NA	Surgery	NA	NA	NA
9	NA	Small intestine	NA	NA	NA	NA	NA	−
10	NA	Small intestine	NA	NA	NA	NA	NA	−
11	68	Small intestine	Nausea and vomiting	5	Surgery	NA	NA	Intraabdominal lymph node
12	62	Stomach	Large ulcer within the stomach	20	Surgery	DOD	7	−
13	20	Colon	Large ulcer within the stomach	6	Chemotherapy	DOD	60	Liver
14	55	Colon	NA	9.5	Surgery	DOD	2	−
15	52	Small intestine	Abdominal pain and vomiting	5	Surgery	AW	13	−
16	70	Duodenum (our case)	NA	1.6	Surgery	AW	10	Intraabdominal lymph node

AW, alive and well; DOD, dead of disease; NA, not available

## Conclusion

In conclusion, a gastrointestinal primary HS recurrence has not been reported previously, and this is the first such case report.

## Data Availability

Not applicable.

## Abbreviations

CT: Computed tomography
HS: Histiocytic sarcoma
MRI: Magnetic resonance imaging
PET: Positron emission tomography
SMA: Superior mesenteric artery
SUV-MAX: Maximum standardized uptake value
